# Defaunation leads to microevolutionary changes in a tropical palm

**DOI:** 10.1038/srep31957

**Published:** 2016-08-18

**Authors:** Carolina S. Carvalho, Mauro Galetti, Rosane G. Colevatti, Pedro Jordano

**Affiliations:** 1 Departamento de Ecologia, Universidade Estadual Paulista (UNESP), 13506-900, Rio Claro, São Paulo, Brazil; 2 Laboratório de Genética & Biodiversidade, ICB, Universidade Federal de Goiás (UFG), Goiânia, Goiás, Brazil; 3 Integrative Ecology Group, Estación Biológica de Doñana, Consejo Superior de Investigaciones Científicas (EBD-CSIC), Sevilla, Spain

## Abstract

Many large species have declined worldwide due to habitat fragmentation and poaching. The defaunation of large frugivores and the consequent reductions of seed dispersal services may have immediate effects on plant demography. Yet, the lasting effects of frugivore defaunation on microevolutionary processes of the plants they disperse remain understudied. We tested if the loss of large seed dispersers can lead to microevolutionary changes of a tropical palm. We show that frugivore defaunation is the main driver of changes in allelic frequency among populations. Turnover of alleles accounted for 100% of dissimilarity in allelic frequencies of individuals between defaunated and non-defaunated forests; and individuals from defaunated sites are 1.5 times more similar genetically than those found in pristine sites. Given that sizeable fractions of the palm fruit crops remain undispersed in defaunated sites due to lack of large-bodied frugivores, this distinct pattern of gene pool composition of early recruits may reveal strong dispersal limitation for specific genotypes, or collapses of gene flow between fragmented areas, or both. Because most of tropical tree species rely on seed dispersal by vertebrates, our results show that defaunation has a lasting effect on microevolutionary processes, with potential consequences for persistence under scenarios of environmental change.

Many species and populations of animals are facing a dramatic decline worldwide, a phenomenon named “Anthropocene defaunation”^1^. The decline of animals may have serious negative ecological and evolutionary consequences, especially if these animals play important roles in mutualistic processes, such as pollination and seed dispersal^2^,^3^,^4^. Vertebrate defaunation frequently targets large-bodied species^1^ and several studies have found that the extinction of seed dispersers may have immediate effects on plant populations, such as significant reductions of fruit removal success^5^, collapse of seed dispersal distance^6^,^7^ and recruitment^8^. Size-biased defaunation of seed dispersers may also impose marked changes in the selective pressures on plants because smaller species cannot provide the same dispersal services, entailing rapid evolutionary changes in seed size^9^. A lasting consequence of these contemporary, human-driven, effects would be changes in the genetic pool caused by the loss of connectivity among fragmented populations due to the reduction of long-distance dispersal (LDD) events^6^,^10^,^11^ and by filtering-out specific genotypes from the gene pool due to the selective foraging behavior of dispersal agents^12^.

Given the negative effects of defaunation on plant populations, we tested the hypothesis that the functional loss of large seed dispersers may lead to microevolutionary changes of the plants that rely on their dispersal. In addition to anthropogenic impacts, the distribution of genetic variability among plant populations over large spatial scales may be also due to a combination of historic and ongoing landscape effects that ultimately influence the joint action of gene flow, selection and genetic drift^13^,^14^,^15^. Therefore, we explored alternative hypotheses (Table 1) defined from specific landscape attributes and used replicated areas in a diverse array of landscape conditions to test the relative importance of alternative drivers of genetic variability distribution^16^.

Here, we analyzed recruits (seedlings) of the palm *Euterpe edulis* in 19 populations in the Brazilian Atlantic forest. This palm is a dominant species distributed across the Atlantic forest of South America^17^. *Euterpe eduli*s produces round fleshy fruits ranging from 8.3 to 14.1 mm in diameter^18^ and they are dispersed by large (e.g., cotingas *Procnia*s *nudicolli*s, toucans *Ramphasto*s spp. and guans *Penelop*e spp. and *Aburria jacuting*a) and medium-sized avian frugivores *(Turdu*s spp.)^9^. We found previously that the functional loss of the large seed dispersers due to defaunation resulted in phenotypic differentiation in seed traits among *E. eduli*s populations and has been a driver for a rapid evolutionary reduction of seed size in defaunated palm populations^9^.

We tested four different hypothetical scenarios to assess variation in local patterns of genetic variability, i.e., to determine which scenario best fits the observed pattern of genetic variation. We thus contrasted genetic variability patterns across replicated local palm populations in different situations according with these scenarios.

## Defaunation Hypothesis

Our working hypothesis is framed on the effects of loss of large-bodied frugivores and the lasting effects of the associated loss of long-distance seed dispersal and reduced recruitment success in defaunated areas^6^,^9^,^10^. Several studies have pointed out the role of seed dispersers in determining the spatial genetic structure of animal-dispersed plants^7^,^13^,^19^,^20^,^21^,^22^. These empirical studies provide convincing evidence of negative impacts of defaunation on tree populations, showing that the foraging behavior and movement pattern of the seed dispersers may affect seed dispersal distance, and consequently, have a lasting signal on the spatial genetic structure of plants at local scales. Large frugivores consume many fruits and a wide range of seed sizes, while smaller birds eat fewer fruits and smaller seeds (<12 mm wide)^9^. Consistent directional selection against large seed size in defaunated areas^9^ may also drive changes in allelic frequencies, with the gene pools of the populations that lost the large seed dispersers being more similar when contrasted with “control” stands in pristine areas, where the full assemblage of mutualistic avian frugivore species is present. Thus, the loss of large seed dispersers may drive microevolutionary changes among *E. edulis* stands due to a selection against large seed size and to the collapse of the long-distance seed dispersal events, impairing effective gene flow (see e.g., Karubian *et al*.^22^, documenting small-scale genetic signatures of variable foraging patterns by frugivores). Simulation models demonstrate that it is possible to find effects of divergent selection on neutral markers, mainly with the combined action of strong selection and very low migration rate^23^. We expect this selection to be strong in defaunated landscapes where the local extinction of large frugivores leads to dramatic decrease in fruit removal rates and seed dispersal effectiveness^10^,^24^.

## Forest Fragmentation

Neotropical forests have been severely affected by habitat loss and fragmentation, resulting in biologically impoverished patches^25^,^26^. Forest fragmentation may lead to drastic reductions in population size and may increase the spatial isolation of populations^27^. Population isolation may raise inbreeding levels by increasing the probability of mating between closely related individuals and self-pollination due to, for example, changes in the composition and behavior of pollinators^28^. Isolation may also limit the dispersal among populations, reducing gene flow and population connectivity^28^. Therefore, we predicted that forest fragmentation, measured by percentage of forest cover, would lead to microevolutionary changes among *E. edulis* populations.

## Biogeographical Origin

The Atlantic forest of South America is heterogeneous in climate, relief and vegetation type^29^ and can be divided into several biogeographical regions^30^. Biogeographical region may also influence the distribution of genetic variability through evolutionary time (phylogeographic effects) and impose distinct selective pressures leading to microevolutionary changes among populations with distinct biogeographical origins. For example, at the community level, plant species composition differs between biogeographical regions composed by rain forest and by semideciduous forest, and plant composition may further include species that are able to cope with a longer dry season^31^. Thus, individuals from the same biogeographical region (here rain forest or semideciduous forest) may share similar gene pool features due to dispersal limitation combined to local adaptation, and we would thus expect microevolutionary changes among individuals from rain forest to semideciduous forest.

## Sampling Design

We sampled the *E. edulis* individuals in delimited and isolated populations with adequate replication across sample groups (strata) defined by the previous hypotheses. We also tested the effects of sampling design as a potential influence for the observed genetic variability distribution, i.e., a situation where differences would emerge by chance effects related to the specific populations sampled.

## Results

**Figure 1:**
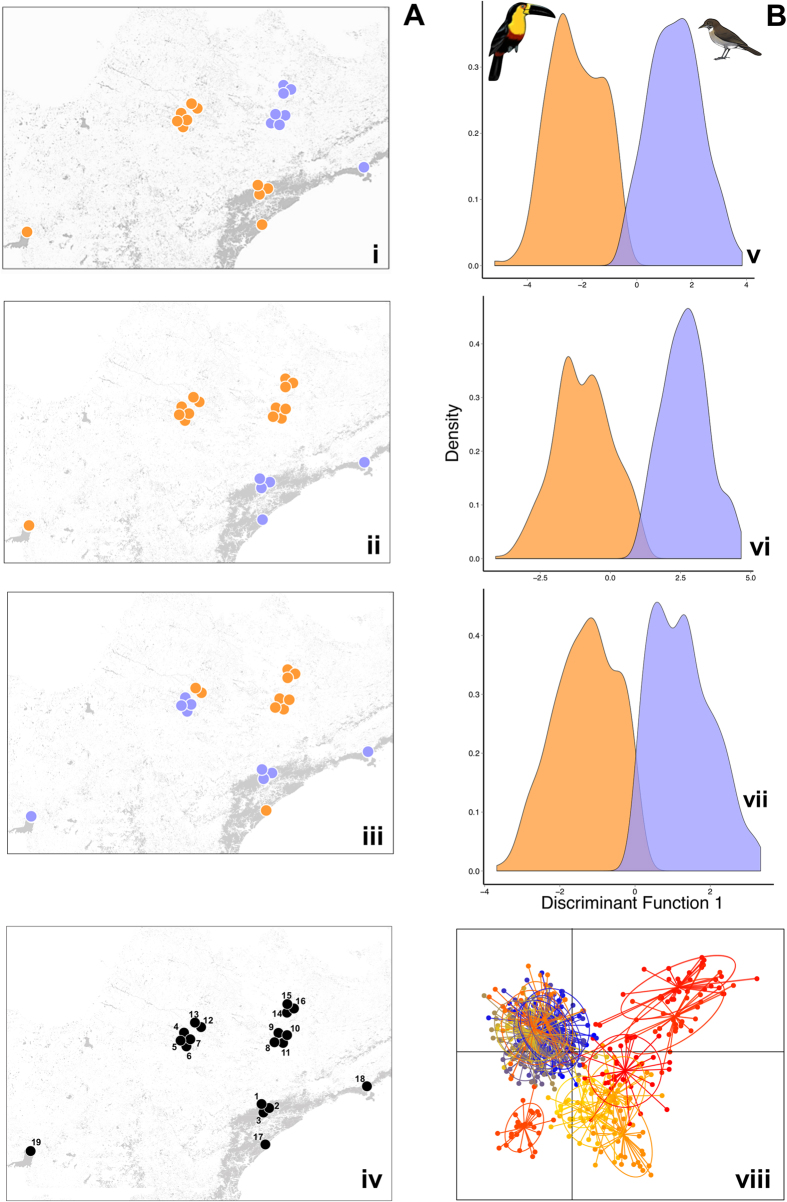
(**A**) Arrangement of sampled sites into groups according to the present-day complexity patterns of the landscape that led to formulation of alternative genetic differentiation hypotheses for *Euterpe edulis* within the Atlantic forest hotspot in Southeast Brazil. Blue and orange colors indicate the groupings of samples being compared under each hypothesis tested: i) Defaunation hypothesis: light blue circles represent sites with functional extinction of large seed dispersers and light orange circles represent sites with the full assemblage of mutualistic avian frugivore species. ii) Biogeographical regions hypothesis: light orange circles represent sites that are in semideciduous forest and light blue circles represent sites that are in rain forest. iii) forest fragmentation hypothesis: light orange circles represent small size sites and light blue circles represent large size sites. iv) Sampling design hypothesis: numbers represent distinct sampling sites. (**B**) Frequency distribution of scores on the first discriminant function for individual genotypes of 545 *Euterpe edulis* seedlings in the Atlantic forest in Southeast Brazil. v) Defaunation hypothesis, vi) Biogeographical regions hypothesis, vii) Forest fragmentation hypothesis, viii) Sampling design hypothesis. The maps were generated using QGIS (www.qgis.org/en/site/) based on a map from SOS Mata Atlântica/INPE (http://mapas.sosma.org.br/). The birds were drawn by Carl Buell.

We found that the hypotheses based on defaunation and biogeographic regions lead to significant microevolutionary changes among populations of *Euterpe edulis* (Table 1, Fig. 1). Both hypotheses had the greatest discrimination power and correct assignment of individual palm genotypes to the *a priori* groupings defined by the hypotheses (represented by kappa index), followed by forest fragmentation and sampling design (Table 1 and Fig. 1). As expected, adscription to biogeographic areas had a significant effect on the differentiation of the genetic pools (kappa estimator of correct assignment, 0.92 [0.88–0.96]). However, the kappa value for the defaunation hypothesis was very similar (0.92 [0.88–0.95]) despite the fact of including populations from either biogeographic origin in each of the groups being compared. The cluster comprising individuals from defaunated populations showed more variability in discriminant function scores when compared to the cluster including individuals from non-defaunated sites and to the clusters related to the hypothesis of biogeographical regions effects (Supplementary Information Figs S1 and S2).

We assessed how defaunation and biogeographical regions have influenced genetic variability and contemporary effective population sizes across the sites. We found that microevolutionary changes between defaunated and non-defaunated sites and between rain forest and semideciduous forest were not due to changes in genetic variability or contemporary effective population size (see Table S4 for genetic variability of each site). Models containing defaunation and biogeographical regions as explanatory variables did not predict variation in genetic diversity (Defaunation: df = 1, F = 0.02, p-value = 0.90; Biogeographical regions: df = 1, F = 1.00, p-value = 0.33), allelic richness (Defaunation: df = 1, F = 2.29, p-value = 0.15; Biogeographical regions: df = 1, F = 0.05, p-value = 0.82), inbreeding coefficient (Defaunation: df = 1, F = 1.14, p-value = 0.30; Biogeographical regions: df = 1, F = 0.57, p-value = 0.46) and contemporary effective population size (Defaunation: df = 1, F = 1.60, p-value = 0.22; Biogeographical regions: df = 1, F = 0.48, p-value = 0.50).

We analyzed whether differences in allelic richness (*β-allelic diversity*) among defaunated and non defaunated sites or among sites from distinct biogeographic regions is due to alleles replacement (turnover, *βsim*) or richness differences driven to passive allelic loss (nestedness-resultant dissimilarity, *βsne*)^32^. The turnover component of allelic richness measures the replacement of alleles in one site by different ones in other site. On the other hand, the nestedness component measures allelic gain or loss; for instance, the alleles of one site with low number of alleles are a subset of alleles of other site containing a high number of alleles^32^. A nested pattern may thus represent two main situations: the alleles missing in populations with lower allelic richness were lost by drift, or the population with higher allelic richness presents ancient alleles that were spread in all populations and new mutations which have not yet widespread. We found that 100% of dissimilarity in allelic richness among palm recruits in defaunated and non defaunated sites (Mean *βdiversity* = 0.64, 95% CI = 0.63 to 0.64; Mean *βsim = *0.64, 95% CI = 0.63 to 0.64; Mean *βsne* = 0.00, 95% CI = 0.00 to 0.00, Supplementary Information Table S5) and among rain forest and semideciduous forest (Mean *βdiversity* = 0.61, 95% CI = 0.60 to 0.62; Mean *βsim* = 0.61, 95% CI = 0.60 to 0.62; Mean *βsne* = 0.00, 95% CI = 0.00 to 0.00, Supplementary Information Table S5) is due to allele turnover. Moreover, we found that palm recruits from defaunated sites are genetically more similar than those from non-defaunated sites; and recruits from rain forests sites are genetically more similar than those from semideciduous forests. The *β*-allelic diversity and its components are lower among palm recruits in defaunated sites (Mean *βsim* = 0.49, SD = 0.10, Mean *βsne* = 0.00, SD = 0.00, Table 1) compared to non defaunated sites (Mean *βsim* = 0.64, SD = 0.09, Mean *βsne* = 0.00, SD = 0.00, Table 1) and lower among individuals in rain forest (Mean *βsim* = 0.53, SD = 0.11, Mean *βsne* = 0.00, SD = 0.00, Table 1) compared to semideciduous forest (Mean *βsim* = 0.58, SD = 0.10, Mean *βsne* = 0.00, SD = 0.00, Table 1).

Finally, we estimated the contribution of spatial effects on genetic differentiation of *E. edulis*. We performed Redundancy Analysis (RDA) using the forward selection method and found that the model including the effects of defaunation and biogeographical regions, but not spatial effects, was the best to explain variation in allele frequencies among sites (*R^2^adj* = 0.12, p-value = 0.01).

## Discussion

Our results indicate that the defaunation of large seed dispersers has a distinct signal on large-scale genetic variability, potentially arising from microevolutionary changes in the palm populations. This supports and extends previous findings^9^ providing evidence for a deeper far-reaching consequence than previously thought. There has been multiple recent evidence demonstrating that the extinction of seed dispersers leads to a decline in recruitment success^8^,^33^ and an increase in plant extinction risk^34^. In addition, previous research has associated seed dispersal limitation with genetic consequences consistent with the loss of gene flow among populations^11^. By assessing realized dispersal (early seedlings established) our study demonstrates for the first time that defaunation may lead to changes in the frequency of alleles among populations, potentially driving long-term homogenization of genetic pools within plant populations from defaunated sites.

We found that defaunation leads to genetic changes in *Euterpe edulis* populations. This could be a synergistic effect from a higher frequency of mating among closely-related individuals, a reduction of effective population size, limited long distance dispersal among populations^11^,^15^,^35^, and rapid evolutionary changes in the seed size of *E. edulis* populations in defaunated areas^9^. Our results suggest that the observed genetic changes were not due to a higher frequency of mating among closely-related individuals or reduction of population size. Instead, the results suggest that genetic changes among *E. edulis* populations were consistently associated with scenarios of strong seed dispersal limitation^9^ and/or collapse of gene flow among fragmented areas^11^. We found that the allelic richness differences among defaunated and non-defaunated sites are largely attributable to allele replacement. Moreover, we found that palm recruits from defaunated sites are genetically more similar than recruits from non-defaunated sites. The altered seed rain composition due to reduction of fruit removal success in defaunated sites^9^ might be determined by distinct combinations of recruited genotypes in these disturbed populations, creating a lasting signal in the composition of the gene pool^12^,^36^. This suggests that strong selection pressure imposed by defaunation could result in changes of allelic frequencies among defaunated and non defaunated sites. Moreover, our results suggest that a similar selective pressure (lack of large-bodied seed dispersers) has led to a homogenization in terms of allelic frequency across palm populations in defaunated sites^37^.

Our results also showed that biogeographical origin has a signature on the genetic structure of *E. edulis* populations, with allele replacement being the main driver of allelic richness differences between rainforest and semideciduous forests. These two biogeographical regions show marked differences in annual rainfall and predominant soil types^38^. There are currently few studies that compare differences in biodiversity composition (genotype, phenotype and species level diversities) between these two forest types^31^,^39^,^40^. Nevertheless, the few available studies show ample differences in plant community composition^31^ among these biogeographical regions. For example, species in semidecidous forest are able to cope with longer dry seasons^31^. Thus, we would expect differences in genetic composition among plant population between rain and semidecidous forest, given the differences among plant community composition between the two biogeographical regions and the potential for a marked phylogeographic signature on genetic diversity. These signals of biogeographical regions on genetic variability distribution can be a result of dispersal limitation within each of the two forest types, genetic drift, and local adaptation of *E. edulis* to these different domains of the Atlantic forest^35^. These historical influences would be “overlaid” on top of the defaunation effects: the DAPC directly contrasting the replicated populations in defaunated and non-defaunated scenarios (i.e., ignoring biogeographic region) indicated that the loss of large dispersers had a distinct signal on genetic structure of *E. edulis* populations, remarkably differentiating the two scenarios despite the variation due to forest physiognomy. Remarkably, the magnitude of this effect appears as large as the magnitude of differentiation when comparing populations from the two biogeographic regions. The large-scale fragmentation and defaunation in Atlantic forest is a recent anthropogenic impact. Despite that, our results reveal that the strong selective pressure imposed by defaunation on the genetic pool of *E. edulis* can be comparable in terms of its effects size with signals left by biogeographical process. If we assume that smaller values of *β*-allelic diversity represent a higher homogenization of genetic pools among populations, ongoing defaunation is driving a stronger genetic homogenization than we would expect just from historical, phylogeographic dynamics.

Our results provide a rigorous test, within an adequately replicated setup of distinct landscape types, of the hypothesis that contemporary defaunation is affecting the diversity of genetic pools of animal-dispersed tropical plants. This supports previous findings^10^,^19^,^33^,^41^,^42^ and strengthens the evidence for pervasive alterations of forest recruitment in disturbed tropical landscapes. Moreover, our results contradict predictions^43^ that rapid genetic changes are not as important as ecological degradation in human-disturbed habitats^15^. Our previous studies showed that the loss of large-bodied frugivores and different biogeographical origin are causing subtle changes in the selection regimes that drive rapid phenotypic evolution in *E. edulis*^9^. We found that this rapid evolutionary change in seed size and limited long distance seed dispersal due to the functional extinction of large frugivores are strong enough to have imprinted the gene pool characteristics of plant populations. These results have broad implications because most tree species are animal dispersed in tropical forests^44^ and defaunation of large frugivores is becoming omnipresent in fragmented and non-fragmented forests^9^. Therefore, the lack of seed dispersers is not only leading to phenotypic changes but also genotypic diversity and gene pool structure, with unknown effects on the long-term persistence of plant species and entire communities. This genetic erosion due to defaunation may be crucial to plants facing ongoing climate change scenarios.

## Material and Methods

### Study sites and species

The Brazilian Atlantic forest is an ideal system to test the effects of antrophocene defaunation on ecological and evolutionary processes. The Atlantic forest originally covered from northeast to south of the Brazilian territory and parts of Argentina and Paraguay^45^, but today it has been reduced to 12% of its original 150 million ha^46^. This reduction entailed a sustained and dramatic process of habitat fragmentation during the last ~500 yr resulting in islands of wild habitat surrounded by crops, pastures and urban matrix and an alarming loss of biodiversity^45^. The Brazilian Atlantic forest can be divided into several biogeographical regions based on climate, altitude and biodiversity composition^30^. Our study covers two of these biogeographical regions: Serra do Mar (here after rain forest) and Interior (here after semideciduous forest). Rain forests occur along the Brazilian Atlantic coast and receive high annual rainfall, whereas semideciduous forests have marked rainfall seasonality and occur in the inland Atlantic forest^47^.

The heart of palm (*Euterpe edulis*, Arecaceae), a threatened Atlantic forest species, was once one of the dominant palms in this ecosystem^17^. The species is present in rain forests and semideciduous forests, where it is restricted to wet microhabitats. Although once abundant, this palm species is currently endangered and locally extinct in many areas owing to illegal harvesting of the edible meristem (heart of palm;^48^). *E. edulis* is a self-compatible monoecious species, but with predominant outcrossed reproduction^49^ and pollination performed mainly by small-sized bees (e.g., *Trigona spinipes*). Their fruits are eaten by more than 58 birds and 20 mammalian species but are dispersed mostly by a reduced subset of large frugivorous birds and thrushes (*Turdus* spp.;^9^,^50^).

### Sampling design and hypotheses for genetic differentiation

We sampled 19 sites from two different biogeographical regions, rain forest and semideciduous forest. The sites had a distinct percentage of forest cover (Supplementary Information Table S1) and well documented bird community composition (Supplementary Information Table S6). Historically, all sites shared a similar assemblage of seed dispersers, but forest fragmentation and hunting have impoverished the assemblage of large frugivores in many sites^9^. We sampled 30 seedlings (total of 545 individuals) of *E. edulis* in each site. To avoid or minimize the effects of spatial genetic autocorrelation, we spaced the sampled seedlings at least 10 m apart. This sampling design allowed us to test distinct hypotheses, once we sampled small and large patches sizes with distinct bird compositions and biogeographical regions (Fig. 1A, see details in Supplementary Information Table S1).

### Genetic analysis

We isolated genomic DNA by using the CTAB extraction procedure and genotyped all individuals using eight microsatellite loci (EE5, EE8, EE25, EE43, EE45, EE47, EE52, EE63), following PCR protocol described by Gaiotto *et al*.^51^. We sized DNA fragments on an ABI Prism 3100 automated DNA sequencer (Applied Biosystems, CA) with the GeneScan ROX 500 size standard (Applied Biosystems, CA), and scored alleles with GeneMapper v4.1 software (Applied Biosystems, CA). We genotyped ten percent of all individuals two times in independent PCR amplifications to check for genotyping error and, we also checked for alleles dropout and null alleles (see details on Supplementary Information Table S2).

### Testing hypotheses for genetic differentiation

We used Discriminant Analysis of Principal Component (DAPC^52^) to find out which hypothesis (defined as *a priori* groupings of the local palm populations according to defaunation status, biogegraphical region, forest fragmentation status and sampling) maximized the differences among groups (clusters). The sampled palm populations were defined according to a replicated design across the different strata defined by each hypothesis (see Fig. 1A). DAPC relies on data transformation using Principal Component Analysis as an *a priori* step to a Discriminant Analysis^52^. DAPC optimizes the separation of individuals into the pre-defined groups^52^. We defined these groups of sampled genotypes according to each of the different hypotheses and ran separate DAPC analyses for each. We used the DAPC to estimate the percentage of correct assignment (proportion of genotypes correctly assigned to their actual sample group) to test how well each hypothesis could discriminate among palm recruits based on grouped genotypes^52^. Thus, we are not using DAPC as an exploratory analysis to find k-clusters, but as a statistical method to test alternative hypotheses about differences among pre-defined groups of samples. To carry out the analysis, we first classified the individuals into groups according to each hypothesis (e.g., samples coming from defaunated sites *vs.* samples from pristine sites). Then, we conducted a DAPC and calculated the proportion of correct assignment of individuals into each hypothesis (defaunation, biogegraphical regions, forest fragmentation and sample design). To avoid over-fitting, we retained 50 principal components, corresponding to 82% of the total genetic information. We carried out the DAPC using the R package *adegenet*^53^. To verify whether the proportion of assignment of each hypothesis differed from a random distribution, we constructed a null model with 1000 permutation and a 95% confidence interval. We also calculated the Kappa estimator, which estimates the mean proportion of corrected assignment after removing correct assignment by chance^54^. We interpreted the hypothesis that had highest proportion of corrected assignment as the most likely hypothesis for distribution of genetic variation.

### Genetic variability and contemporary effective population size

We assessed the influence of defaunation and biogegraphical regions on genetic variability and contemporary effective population size across the study sites. We estimated genetic diversity (*H*_*e*_ - expected heterozygosity under Hardy-Weinberg equilibrium, following Nei^55^), allelic richness based on rarefaction analysis (*AR*^56^) and inbreeding coefficient (*f*, obtained from analysis of variance of allelic frequency^57^) using the software FSTAT 2.9.3.2^58^. We also estimated contemporary effective population size (*Ne*), using the software NeEstimator^59^. To test whether defaunation and biogegraphical regions are correlated with variation in genetic variability and contemporary effective population size, we conducted a generalized linear model (GLM), after testing for spatial autocorrelation (see details on Supplementary Information Table S3).

### Turnover component of allelic richness

The difference in allelic richness among sites may be mainly due to allele replacement (turnover) or richness differences (nestedness-resultant dissimilarity^32^). We estimated each component using the R package *betapar*t^60^, which partitions allelic richness dissimilarity into turnover and nestedness components. In contrast to Diniz-Filho *et al*.^32^ that estimated these components using presence/absence of alleles, we carried out the analysis using allelic frequency. We used the Bray-Curtis’s dissimilarity index to estimate *β*-allelic diversity and its components, and we also calculated the relative importance of the turnover component for the overall allelic richness dissimilarity. We carried out the *β*-allelic diversity analysis in two ways. First, we randomly sampled all palm recruits from one site of each group of each hypothesis (i.e. one from defaunated and other from non-defaunated sites or one from rain forest and other from semideciduous forest) and estimate the *β*-allelic diversity and its components among these groups (i.e. *β*-allelic diversity between defaunated and non-defaunated sites or between rain forest and semideciduous forest). This procedure were carried out 499 times (bootstrapping) and we obtained the mean, standard deviation and 95% confidence interval for each *β* diversity component. We constructed a null model with 1000 permutation and a 95% confidence interval to verify whether the results of the *β* diversity analysis for each hypothesis differed from random. With this analysis we could estimate the importance of each *β*-allelic diversity component for the overall allelic richness dissimilarity between defaunated and non-defaunated sites or between rain forest and semideciduous forest. Second, we randomly sampled all palm recruits from two sites of each group (i.e. two sites from defaunated sites) and estimate the *β*-allelic diversity and its components among the sites from each group. We carried out this procedure 499 times (bootstrapping) and obtained the mean, standard deviation and 95% confidence interval for each *β* diversity component. The same was done for all groups of all hypotheses (defaunated, non-defaunated, rain forest and semideciduous forest). With this analysis we could compare and contrast the *β*-allelic diversity results between defaunated and non-defaunated sites or between rain forest and semideciduous forest. For example, we expected that *β* diversity might be lower among individuals in defaunated sites compared to non-defaunated sites.

### Spatial component of genetic differentiation

The spatial distribution of individuals and populations are also important factors that affect genetic differentiation patterns. For example, some populations can follow an isolation by distance model while others may follow an island model. To deal with spatial effects on genetic differentiation, we conducted a Redundancy Analysis (RDA). RDA is a multivariate method that assesses the influence of a matrix of independent variables (e.g. geographic coordinates) on a matrix of dependent variables (e.g. alleles frequency)^61^. RDA also allows the use of environmental data as independent variables^61^. We performed a RDA using an allele frequency matrix as the response variable and the spatial variable, defaunation and biogeographical regions as the explanatory variables. For RDA model selection, we performed RDA using the forward selection method with double stop criteria of Blanchet^62^. To reduce the incorporation of too many variables into the model and to not inflate type I error, Blanchet *et al*.^62^ proposed a forward selection based on two criterion: the forward selection is stop if either the significance level alpha is reached or the global adjusted coefficient of multiple determination (*R^2^*_*adj*_) is exceeded. We used the *adegenet* R package^53^ to calculate allele frequencies for each site and *packfor*^63^ and *vegan*^64^ R package to carry out RDA and forward selection.

## Additional Information

**How to cite this article**: Carvalho, C. S. *et al*. Defaunation leads to microevolutionary changes in a tropical palm. *Sci. Rep.* **6**, 31957; doi: 10.1038/srep31957 (2016).

## Supplementary Material

Supplementary Information

Supplementary Dataset 1

## Figures and Tables

**Table 1:** Correct assignment statistics and *β* diversity analysis of individuals of *Euterpe edulis* in Atlantic forest in Southeast Brazil, into groups according to different hypotheses driving genetic differentiation.

Hypothesis	Predictions	Kappa [CI]	*β*-allelic diversity [SD]
**Defaunation:** The loss of large seed dispersers may led to microevolutionary changes among populations due to a selection against large seed size and to the collapse of the long-distance seed dispersal events.	High correct assignment (Kappa) and higher *β*-allelic diversity among non-defaunated than defaunated sites	0.92[0.88–0.95]	Defaunated sites: 0.49 [0.10]
			Non defaunated sites: 0.64 [0.09]
**Biogeographical regions:** Rain forest and semideciduous forest may led to microevolutionary changes among populations due to their influence on the distribution of genetic variability through evolutionary time, and due to the distinct selective regimes imposed by biogeographical differences between the two regions.	High correct assignment (Kappa) and *β*-allelic diversity among rain forest sites similar to semideciduous forest sites	0.92[0.88–0.96]	Rain forest sites: 0.53 [0.11]
			Semideciduous forest sites: 0.58 [0.10]
**Forest Fragmentation:** Forest fragmentation may led to microevolutionary changes among populations because of the associated drastic reductions in population size, and may also increase the effect of spatial isolation among populations.	High correct assignment (Kappa)	0.75[0.69–0.80]	—
**Sampling design:** Given that individuals were sampled in delimited and isolated populations, the sampling design may generate differences in the gene pool among populations.	High correct assignment (Kappa)	0.58[0.54–0.62]	—

Hypothesis - hypothesis for main drivers of microevolutionary changes; Predictions - predictions for main drivers of microevolutionary changes; Kappa - kappa estimator for correct assignment, the larger is the kappa estimator, the greater is the support of the data for a hypothesis; CI95% Confidence Interval; *β*-allelic diversity - *β*-allelic diversity using Bray-Curtis’s dissimilarity index; SD - Standard Deviation
