# Supplementary material for: Defaunation leads to microevolutionary changes in a tropical palm
Source: Sci Rep. 2016 Aug 18;6:31957. doi: 10.1038/srep31957 (PMC4989191; doi:10.1038/srep31957)
Supplement: Supplementary Information [file srep31957-s1.pdf]

Supplementary Information

Defaunation leads to microevolutionary changes in a tropical palm

Carolina S. Carvalho, Mauro Galetti, Rosane Colevatti, Pedro Jordano

Sampling design and hypotheses for genetic differentiation

We sampled 19 sites from different biogeographical regions (rain forest and semideciduous forest sites), with distinct forest cover and well known bird community composition. We subdivided the sites into small and large patch size. To be considered in the small patch size category, sites should have less than 200 ha and a percentage of forest cover within a radius of 500 m lower than 30% (threshold in forest amount to severe species loss, low genetic diversity and high genetic differentiation). On the other hand, to be included into the category of large patch size, sites should have more than 500 ha and percentage of forest cover above 40%. Patch size was measured using the mapping with visual digitalization and classification at the scale of 1:5,000 using high-resolution 1x1 m images available at Google Earth (http://earth.google.com). QGIS (www.qgis.org) software was used to access the images on the fly with the OpenLayer plugin (OpenLayer 2013).

We classified sites into defaunated and non-defaunated categories. We considered as defaunated sites those which present functional loss of large frugivores (mean grape width > 12 mm following Galetti et al. 2013). The data on frugivore community composition were obtained from Galetti et al. 2013, the WikiAves dataset (www.wikiaves.com) and personal observation. We used the classification of Veloso to separate the sites into rain forest and semideciduous forest. Table S1 shows the characteristics of each site.

Table S1 Sampling location for the 19 sites of *Euterpe edulis* in Atlantic Forest in Southeast Brazil. N, number of individuals sampled. Defaunation status – Defaunated = fuctional loss of large frugivores. Habitat loss status – small patch = Patch size < 200 ha and landscape forest cover <

30%, large patch = Patch size > 500 ha and landscape forest cover > 40%. Biogeographical regions – Rain forest, Semideciduous forest.

Sites	N	Defaunation status	Fragmentation status	Biogeographical regions
1	30	Non-Defaunated	Large patch	Rain forest
2	30	Non-Defaunated	Large patch	Rain forest
3	30	Non-Defaunated	Large patch	Rain forest
4	30	Non-Defaunated	Large patch	Semideciduous forest
5	30	Non-Defaunated	Large patch	Semideciduous forest
6	30	Non-Defaunated	Large patch	Semideciduous forest
7	31	Non-Defaunated	Large patch	Semideciduous forest
8	14	Defaunated	Small patch	Semideciduous forest
9	28	Defaunated	Small patch	Semideciduous forest
10	28	Defaunated	Small patch	Semideciduous forest
11	30	Defaunated	Small patch	Semideciduous forest
12	30	Non-Defaunated	Small patch	Semideciduous forest
13	30	Non-Defaunated	Small patch	Semideciduous forest
14	31	Defaunated	Small patch	Semideciduous forest
15	31	Defaunated	Small patch	Semideciduous forest
16	30	Defaunated	Small patch	Semideciduous forest
17	28	Non-Defaunated	Small patch	Rain forest
18	28	Defaunated	Large patch	Rain forest
19	26	Non-Defaunated	Large patch	Semideciduous forest

Genetic analysis

All pairs of microsatellites loci were in linkage equilibrium (all $p > 0.05$) and there was no evidence of genotyping errors or null alleles (results not shown). All loci presented high genetic variability, but the observed heterozygosity differed from the expectation under Hardy-Weinberg equilibrium for all loci (all $p < 0.001$, Table S2).

Table S2 Genetic characterization of the eight microsatellite loci used in this study, based on 545 individuals of *Euterpe edulis* from 19 sites in Atlantic Forest remnants in Southeast Brazil. A, number of alleles; H_e, expected heterozygosity; H_o, observed heterozygosity; F_{IS}, inbreeding coefficient (* significant, $p < 0.001$); SD, standard desviation.

Locus	*A*	H_e	H_o	F_{IS} (SD)
**EE5**	25	0.668	0.527	0.218 (0.047)*
**EE8**	22	0.728	0.615	0.161 (0.037)*
**EE43**	13	0.737	0.564	0.226 (0.057)*
**EE52**	25	0.874	0.661	0.254 (0.052)*
**EE63**	19	0.738	0.643	0.118 (0.041)*
**EE25**	23	0.853	0.678	0.218 (0.048)*
**EE47**	24	0.750	0.684	0.102 (0.032)*
**EE45**	19	0.745	0.509	0.327 (0.048)*
**Overall loci**		0.762	0.610	0.197 (0.025)*

Genetic variability and contemporary effective population size

To account for the presence of autocorrelation, we tested models with distinct spatial covariance structures (Gaussian, Exponential and Spherical) using the restricted maximum likelihood (REML) method in a GLM and compared with a model without spatial covariance structure. These models contained defaunation and biogeographical regions classification as explanatory covariates. To find the best spatial covariance structure, we compared the models using Akaike Information Criteria (AIC). Models with ΔAICc< 2 were considered as equally plausible to explain the observed pattern.

Table S3 Model selection of model with distinct spatial covariance structures (Gaussian, Exponential and Spherical) and a model without spatial covariance structure for H_e (expected heterozygosity – genetic diversity), *AR* (allelic richness), F_{IS} (inbreeding coefficient) and *Ne* (contemporary effective population size) in 19 sites of *Euterpe edulis* in Atlantic forest remnants in Southeast Brazil. These models contained defaunation and biogeographical regions classification as explanatory variables. *K*, number of parameters estimated for each model; ΔAICc, Akaike corrected for small samples

	H_e		AR		f		Ne	
	**K**	**ΔAICc**	**K**	**ΔAICc**	**K**	**ΔAICc**	**K**	**ΔAICc**
Without spatial covariance structure	5	0.0	5	0.0	5	0.0	5	0.0
Exponential	6	4.4	6	4.4	6	4.4	6	4.9
Gaussian	6	4.4	6	4.4	6	4.4	7	4.9
Spherical	6	4.4	6	4.4	6	4.4	7	4.9

Table S4 Genetic variability in 19 sites of *Euterpe edulis* in Atlantic forest remnants in Southeast Brazil. N – number of sampled individuals; H_e - expected heterozygosity; H_o - observed heterozygosity; AR - allelic richness; F_{IS} - inbreeding coefficient; Ne - contemporary effective population size.

Site	N	H_e	H_o	AR	F_{IS}	Ne
1	30	0.766	0.564	8.8	0.264	310.0
2	30	0.808	0.571	8.6	0.295	209.2
3	30	0.787	0.591	8.6	0.248	Inf
4	30	0.815	0.695	9.0	0.148	40.3
5	30	0.781	0.702	7.5	0.101	35.8
6	30	0.786	0.631	9.2	0.197	767.3
7	31	0.799	0.651	7.4	0.186	20.7
8	14	0.864	0.661	9.0	0.235	143.0
9	28	0.736	0.631	6.9	0.143	48.5
10	28	0.799	0.627	8.3	0.216	61.4
11	30	0.729	0.597	6.3	0.182	3.1
12	30	0.794	0.633	8.6	0.203	18.4
13	30	0.724	0.629	7.5	0.131	17.8
14	31	0.716	0.551	6.2	0.229	19.5
15	31	0.724	0.524	6.4	0.275	6.9
16	30	0.816	0.581	8.3	0.288	22.6
17	28	0.654	0.602	7.0	0.079	Inf
18	28	0.691	0.585	6.7	0.153	50.4
19	26	0.677	0.560	6.0	0.172	25.5

Testing distinct hypotheses for genetic differentiation

R code:

```
## 1. Calculate proportion of observed/predicted assingment
Hypothesis1 <- dapc(data, paste(groups$M1), n.pca=50, n.da=2)
Hypothesis1.summary <- summary(Hypothesis1)
Hypothesis1.assign <- Hypothesis1.summary$assign.prop

## 2. Calculate proportion of random assingment using 1000 permutations
Hypothesis1.aleat<-numeric(1000)
for(i in 1:1000){
  groups.aleat<-sample(groups$M1)
  Hypothesis1.aleat <- dapc(data, grupo.aleat, n.pca=50, n.da=2)
  Hypothesis1.aleat.summary <- summary(Hypothesis1.aleat)
  Hypothesis1.aleat.assign <- Hypothesis1.aleat.summary$assign.prop
  Hypothesis1.aleat[i] <- Hypothesis1.aleat.assign
}

## 3. Calculate 95% CI
mean.Hypothesis1.aleat <- mean(Hypothesis1.aleat)
sd.Hypothesis1.aleat <- sd(Hypothesis1.aleat)
CIinf.Hypothesis1.aleat <- mean.Hypothesis1.aleat-(1.96*(dp.Hypothesis1.aleat /sqrt(1000)))
CISup.Hypothesis1.aleat <- mean.Hypothesis1.aleat+(1.96*(dp.Hypothesis1.aleat /sqrt(1000)))
```

Table S5 Correct assignment of individuals of *Euterpe edulis* in Atlantic Forest in Southeast Brazil, into different hypothesis driving genetic differentiation. % Correct assignment, percentage of corrected assignment using Discriminant analysis of Principal Components; % Random assignment, percentage of corrected assignment of null model using Discriminant analysis of Principal Components; Bray-Curtis Null model, Bray-Curtis's dissimilarity index of null model; Turnover Null model, turnover dissimilarity component of null model; Nestedness Null model, nestedness-resulting dissimilarity component of null model; CI, Confidence Interval.

Hypothesis	% Correct assignment	% Random assignment [CI]	Bray-Curtis Null model [CI]	Turnover Null model [CI]	Nestedness Null model [CI]
Defaunation	0.96	0.65	0.59	0.59	0.00

Hypothesis	% Correct assignment	% Random assignment [CI]	Bray-Curtis Null model [CI]	Turnover Null model [CI]	Nestedness Null model [CI]
		[0.65 - 0.65]	[0.58 – 0.60]	[0.58 – 0.60]	[0.00 – 0.00]
Biogeographical regions	0.97	0.74 [0.74 - 0.74]	0.59 [0.58 – 0.60]	0.59 [0.58 – 0.60]	0.00 [0.00 – 0.00]
Defaunation + Biogeographical regions	0.95	0.50 [0.49 – 0.50]	-	-	-
Fragmentation	0.87	0.62 [0.62 - 0.63]	-	-	-
Population	0.60	0.21 [0.21 - 0.21]	-	-	-

Figure S1. First discriminant function scores of individual genotypes from 545 seedlings of *Euterpe edulis* in Atlantic Forest in Southeast Brazil. Light orange boxplots represent individuals into sites with functional extinction of large seed dispersers and light blue boxplots represent individuals into sites with the full assemblage of mutualistic avian frugivore species. The center vertical line is the median of the sample. The top of the box above the median shows the 75th percentile and the bottom of the box bellow the median shows the 25th percentile. The whiskers show the maximum and minimum values of the sample.

Figure S2. First discriminant function of individuals genotypes of 545 seedling of *Euterpe edulis* in Atlantic Forest in Southeast Brazil. Light orange boxplots represent individuals into sites that are in semideciduous forest and light blue boxplots represent individuals into sites that are in rain forest. The center vertical line is the median of the sample. The top of the box above the median shows the 75th percentile and the bottom of the box bellow the median shows the 25th percentile. The whiskers show the maximum and minimum values of the sample.

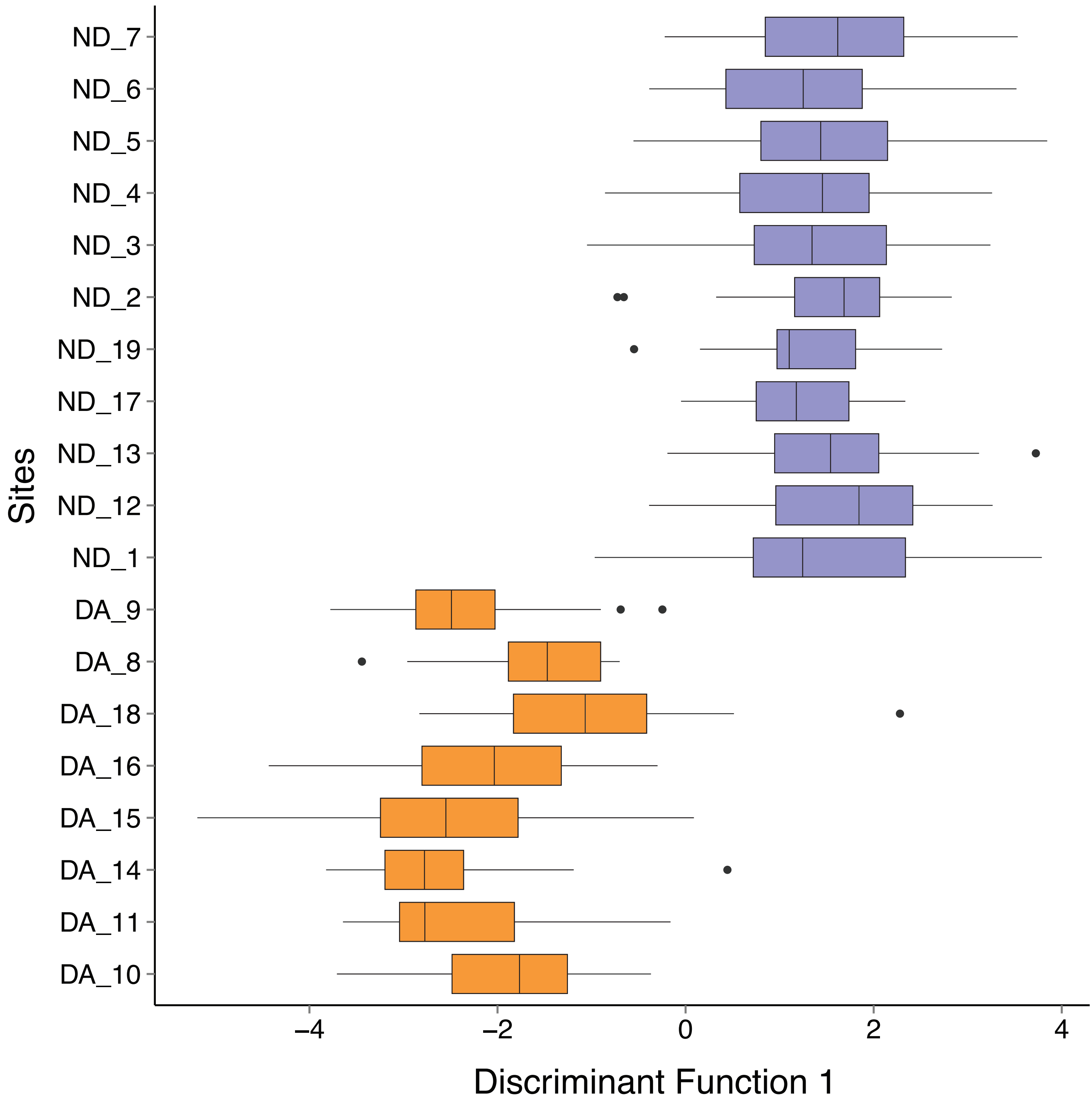

ND_7
ND_6
ND_5
ND_4
ND_3
ND_2
ND_19
ND_17
ND_13
ND_12
ND_1
DA_9
DA_8
DA_18
DA_16
DA_15
DA_14
DA_11
DA_10
Sites
−4
−2
0
2
4
Discriminant Function 1

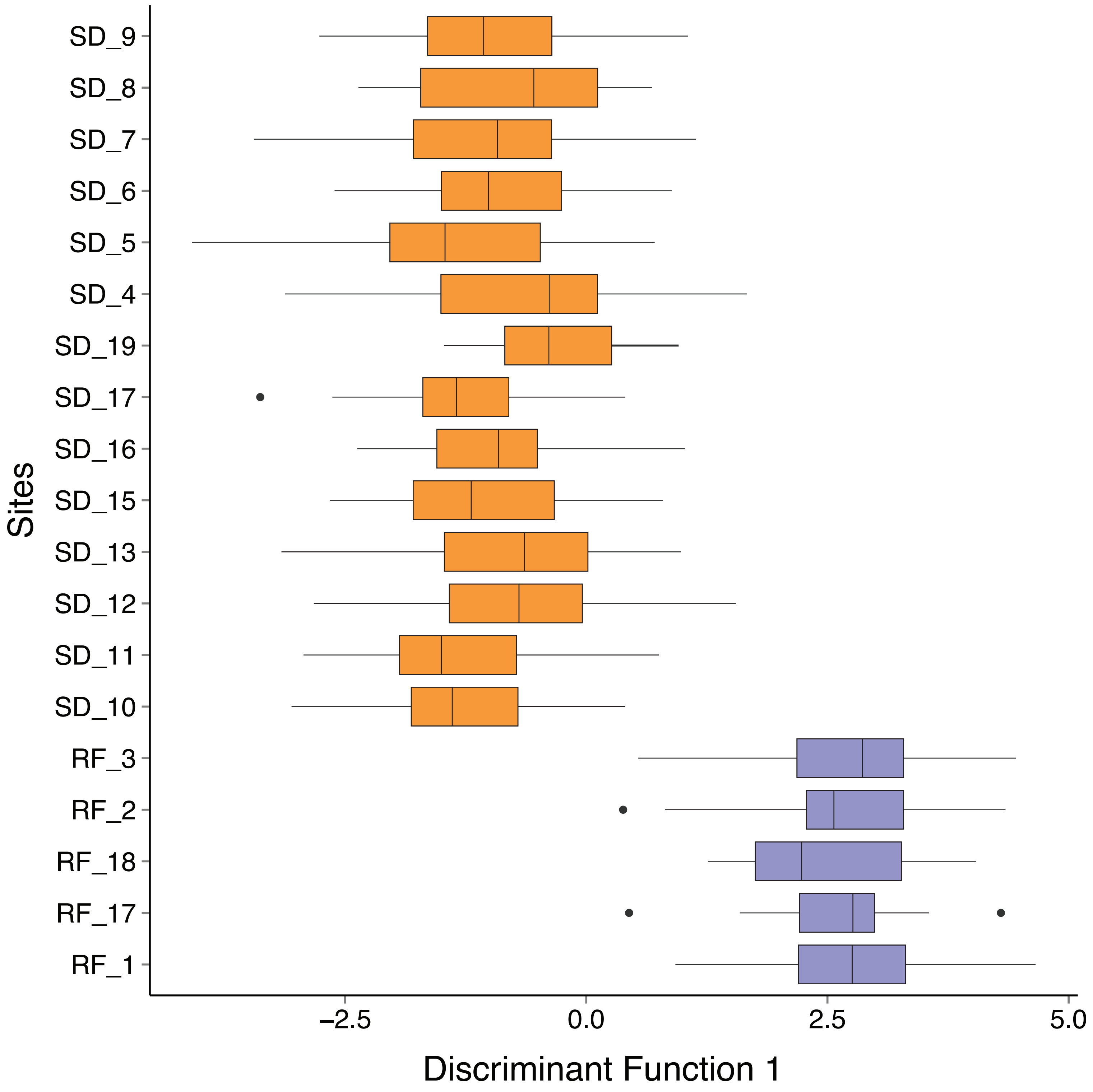

Sites
SD_9
SD_8
SD_7
SD_6
SD_5
SD_4
SD_19
SD_17
SD_16
SD_15
SD_13
SD_12
SD_11
SD_10
RF_3
RF_2
RF_18
RF_17
RF_1
−2.5
0.0
2.5
5.0
Discriminant Function 1
